# Evaluation of Antioxidant Activities and Tyrosinase Inhibitory Effects of *Ginkgo biloba* Tea Extract

**DOI:** 10.1155/2022/4806889

**Published:** 2022-03-16

**Authors:** Pongsathorn Klomsakul, Arisara Aiumsubtub, Pornchanok Chalopagorn

**Affiliations:** ^1^Biology Program, Faculty of Science and Technology, Phranakhon Rajabhat University, Bangkok 10220, Thailand; ^2^Chemistry Program, Faculty of Science and Technology, Phranakhon Rajabhat University, Bangkok 10220, Thailand

## Abstract

*Ginkgo biloba* L. (*Ginkgoaceae*) is one of the best-selling products, popular in nutritional properties and health benefits. In the present study, the total phenolic compounds and flavonoid content of the ethanolic extract from *G. biloba* tea were also evaluated. Furthermore, the antioxidant activity was determined using DPPH assay and tyrosinase inhibitory activity was also determined with L-DOPA as a substrate. The extract showed the total phenolic compound and flavonoid content were 14.13 mg GE g^−1^ extract and 71.33 mg rutin equivalence g^−1^ DW, respectively. Taking into account the results of the DPPH, the antioxidant property at the concentration of 500 *µ*g ml^−1^ was 95.29% that is similar to that of the BHT, ascorbic acid, and gallic acid used as positive controls. The inhibitory capacity of the sample against tyrosinase is lower than that of positive controls at all concentrations. The results of inhibition in terms of IC_50_ confirm the inhibition patterns. On the other hand, the statistical similarity of the anti-DOPA auto-oxidation (IC_50_) of *G. biloba* leaf *extract* and kojic acid was found (456.27 and 418.5 *µ*g ml^−1^) but was lower than that of ascorbic acid (IC_50_ 989.61 *µ*g ml^−1^). A relationship was observed between the potential of antioxidant activity, tyrosinase inhibition, and anti-DOPA auto-oxidation with concentration levels of the extracts. The results of phytochemical analysis revealed the presence of tannins, flavonoids, terpenoids, and reducing sugars.

## 1. Introduction

*Ginkgo biloba* L. belonging to the Ginkgoaceae family, more commonly known as ginkgo or maidenhair tree, is the most ancient living gymnosperm and native to China. It has been widely used therapeutically in traditional Chinese medicine for centuries. *G. biloba* is a dietary supplement promoted for sharpening memory and improving circulation [1]. At present, both the seeds and leaves are recommended for treating heart and lung problems. Moreover, *G. biloba* extract is used for the treatment of peripheral vascular disease and cerebrovascular insufficiency in the elderly; in particular, intermittent claudication and symptoms thought to be due to cerebral insufficiency (difficulties of concentration and memory, confusion, dizziness, tinnitus) are reported to be relieved by the extract without side effects, and antioxidant action has been claimed [2]. Ginkgo leaves can be used to make tea, herbal extracts, tinctures, and pills. The major bioactive compounds of *G. biloba* are reported to be terpenoids, flavonoids, biflavonoids, organic acids, polyphenols, and others. The ginkgolides A, B, and C and sesquiterpene bilobalide are the major constituents of *G. biloba* that exhibit biological and pharmacological activities [3, 4].

Antioxidant activity is a very important pharmacological property. Many pharmacological functions such as antiaging, antimutagenicity, anticarcinogenicity, and skin whitening originated from this property [5]. Numerous spices of medicinal plants have been tested in an effort to identify novel and potential antioxidants [6, 7]. Studies of natural antioxidants are coming to front to researchers for use in foods or medicinal materials to replace synthetic antioxidants. Particularly, recent studies are focused on natural products including vegetables and wild plant sources [8].

In the production of melanin, tyrosinase (E.C. 1.14.18.1) is a copper enzyme that catalyzes both the monophenolase and diphenolase activities. Tyrosinase first catalyzes the hydroxylation of L-tyrosine to L-3,4-dihydroxyphenylalanine (L-DOPA). Second, L-DOPA oxidation to L-DOPA-quinone is catalyzed [9]. Quinone is a very reactive component. It can be converted into melanin through a sequence of nonenzymatic processes [10]. Generating tyrosinase blockers will not only address the need for skin-whitening medicines but also provide the groundwork for medications to treat discoloration. Furthermore, it has been reported that tyrosinase might contribute to the dopamine neurotoxicity and neurodegeneration associated with Parkinson's disease [11]. Medicinal plant extracts have received increased attention in recent years for use in the development of contemporary medical and cosmetic goods. Numerous researchers have advocated for a greater usage of plant-based natural medicinal products [12]. Several plant-derived compounds have been explored as cosmetics and medicines to inhibit melanin oversupply as whitening agents. A variety of variables can influence skin whitening. It is thought that antioxidant capabilities had a significant effect on suppressing melanin production [13].

Therefore, the objective of this research is to extract the substances in *G. biloba* tea and determine the total phenolic compounds and flavonoid content. These compounds have been reported that they are antioxidants or inhibitors of some enzymes [14, 15], antioxidation activity *in vitro* tyrosinase inhibition and anti-DOPA auto-oxidation effects were evaluated.

## 2. Materials and Methods

### 2.1. Chemicals and Reagents

Ascorbic acid, aluminum chloride, 3,4-dihydroxy-l-phenylalanine, 1,1-diphenyl-2-picrylhydrazyl, Folin–Ciocalteu reagent, gallic acid, lyophilized mushroom tyrosinase, sodium carbonate, ferric chloride, ethyl acetate, chloroform, formic acid, acetonitrile, and water (HPLC grade) were purchased from Sigma-Aldrich Chemie, Germany. Ethanol was purchased from Merck, Germany. Standards of bilobalide, ginkgolide A, and ginkgolide B were purchased from ChemFaces, China.

### 2.2. *G. biloba* Extraction

The dried *G. biloba* L. leaves were processed at the tea factory of Herb's Friend Farming House Wife Group of Nakornprathom Province in Thailand (Pol. Lt. Col. Chariya Sandach). Dried *G. biloba* leaves were grinded into powder. For extraction, 80% ethanol was used to extract the soluble compounds from the *G. biloba* L. leaves. The dried leaf powder (5 g) was mixed with ethanol (1 : 20, *w*/*v*) at room temperature for 72 h, and the extraction was repeated three times. The extracts were filtered and evaporated using a vacuum rotary evaporator. The extract obtained was then transferred to vials and kept in the dark at −20°C prior to use.

### 2.3. Determination of Total Phenolics

The total phenolic content of the crude extract was determined calorimetrically using the Folin–Ciocalteu method [16]. To achieve this purpose, 2.0 ml (1.0 mg ml^−1^ in H_2_O Milli-Q) aliquots of the fractions were added to 2.5 ml of recently prepared Folin–Ciocalteu reagent, followed by the addition of 5 ml of an aqueous 14% sodium carbonate solution. The mixture was stirred and incubated at room temperature for 2 h. The absorbance at 760 nm was measured using a UV/VIS spectrophotometer. Gallic acid was used as a standard to produce the calibration curves. The results were expressed in mg of gallic acid equivalents (GAE) g^−1^ of dry sample.

### 2.4. Determination of Flavonoid Content

The total flavonoid content was determined [17]. Briefly, 5 ml of 2% aluminum trichloride (AlCl_3_) in ethanol was mixed with the same volume of *G. biloba* tea extract solution. Absorption readings at 415 nm (UV/VIS spectrophotometer) were taken after 10 min against a blank sample consisting of a 5 ml extract solution with 5 ml ethanol without AlCl_3_. The total flavonoid content was determined using a standard curve with rutin (0–50 mg l^−1^) as the standard. The mean of three readings was used and expressed as mg of rutin equivalents (RE) g^−1^ of dry weight.

### 2.5. Scavenging Activity of DPPH Radical

The scavenging activity of the extract on 1,1-diphenyl-2-picrylhydrazyl (DPPH) was determined according to the method with slight modification [18]. 0.1 ml of samples (1 mg ml^−1^) or standard antioxidant solution (vitamin C) was added to an ethanolic solution (0.9 ml, 1.5 × 10^−4^ M) of DPPH radical. The mixture was shaken vigorously and kept within the dark at room temperature for 30 min, and the absorbance of the solution was then measured spectrophotometrically at 517 nm. The reduction in the absorbance of the DPPH solution indicated the free radical scavenging activities of those antioxidants. The DPPH radical scavenging activity was calculated according to the following formula:

(1) $$\begin{array}{c} \text{scavenging rate}\left(\%\right)=\frac{\left(1-A\right)}{A_{0}}\times 100, \end{array}$$

where *A*_0_ is the absorbance of the control reaction (containing all reagents except the tested compound) and *A* is the absorbance of the tested compound.

### 2.6. Determination of Tyrosinase Inhibition

The determination of tyrosinase was performed using L-DOPA as a substrate according to the method described before with slight modification [19, 20]. First, 0.8 ml of 2.5 mM L-DOPA was mixed with 2.4 ml of 50 mM sodium phosphate buffer (pH 6.5) and incubated at 37°C for 10 min. Then, 0.15–2.5 ml of 2 mg ml^−1^ samples and 1.0 ml tyrosinase solution were added in this order to the mixture. The IC_50_ value of samples was determined at a concentration of 62.5–1000 *μ*g/ml. This solution was immediately monitored for dopachrome formation by measuring the absorption at 475 nm for 5 min. Triplicate measurements were recorded. The inhibition of tyrosinase activity was calculated according to the following formula:

(2) $$\begin{array}{c} \text{Tyrosinase inhibition}\left(\%\right)=\frac{\left(C-D\right)-\left(A-B\right)}{C-D}\times 100, \end{array}$$

where *A* is with sample and L-DOPA, *B* is with sample without L-DOPA, *C* is with substrate without sample, and *D* is without substrate and sample.

### 2.7. Determination of L-DOPA Auto-Oxidation Inhibition

*G. biloba* tea extracts (0.1–10000 *µ*g ml^−1^) were used to test the inhibition activity. 0.5 ml of 0.5 mM DOPA in 50 mM sodium phosphate buffer (pH 6.5) was mixed with 0.5 ml of the extracts for a total volume 500 *µ*l of the assay mixture. The mixture was incubated at 30°C for 48 h. The inhibition activity was measured at wavelength 475 nm. The half-maximal inhibitory concentration (IC_50_) was calculated [21].

### 2.8. Phytochemical Qualitative Analysis

The phytochemical analysis of *G. biloba* tea was performed using the following methods [22].

#### 2.8.1. Test for Tannins

0.5 g of sample was boiled in 10 ml of distilled water and filtered. A few drops of 0.1% ferric chloride were added, and the formation of brownish green or blue black color indicated the presence of tannins.

#### 2.8.2. Test for Saponins

1 g of sample was boiled in 10 ml of distilled water and filtered. 5 ml of distilled water was added and shaken vigorously. A few drops of olive oil were mixed and shaken vigorously again. The foam appearance showed the presence of saponin.

#### 2.8.3. Test for Flavonoids

1 g of sample was boiled in 10 ml of ethyl acetate for few minutes and filtered. 5 ml of the filtrate was shaken with 1 ml of 10% ammonia solution. The formation of yellow color indicated the presence of flavonoids.

#### 2.8.4. Test for Terpenoids

2 ml of chloroform was added with the 5 ml aqueous sample. 2 ml concentrated H_2_SO_4_ was carefully added to a layer. The formation of reddish brown color indicated the presence of terpenoids.

#### 2.8.5. Test for Steroids

2 ml of acetic anhydride and 2 ml concentrated H_2_SO_4_ were added to 0.5 g of sample. The color change to blue or green indicated the presence of steroids.

#### 2.8.6. Test for Anthraquinones

5 ml of chloroform was added to 0.5 g of sample. The mixture was shaken for 5 min and filtered. 10% ammonium solution was added and shaken. The formation of bright pink color in the aqueous layer indicated the presence of anthraquinones.

#### 2.8.7. Test for Reducing Sugars

1 g of sample was boiled in 10 ml of distilled water and filtered. A few drops of 20% sodium hydroxide solution and an equal volume of Benedict solution were added. The mixture solution was boiled for 3 min. The formation of brick red color indicated the presence of reducing sugar.

### 2.9. HPLC Analysis

The HPLC analysis of *G. biloba* extract was carried out using a chromatographic system (Agilent 1100 series HPLC system). The column used was Eurospher 100-5 C18 (Knauer; 250 × 4 mm, 5 *µ*m). The eluent was formic acid (0.3%) to acetonitrile (70 : 30 *v*/*v*), and the separations were performed by using isocratic mode, elution performed at a flow rate of 0.85 ml/min. The detection was done at 219 nm by using a UV detector. All chromatographic data were recorded and processed using CAG Bootp server software.

### 2.10. Statistical Analysis

Comparison of the means of three replicate determination ± standard deviation was done. The statistical analysis of data was performed using SPSS 22 for Windows. To determine whether there have been any differences between activities of samples, variance analysis was applied to the result. *p* ≤ 0.05 was considered as a significant difference (*α* = 0.05).

## 3. Results and Discussion

### 3.1. Ethanolic Extract, Total Phenolic Compounds, Flavonoid Content, and DPPH Assay

**Figure 1:**
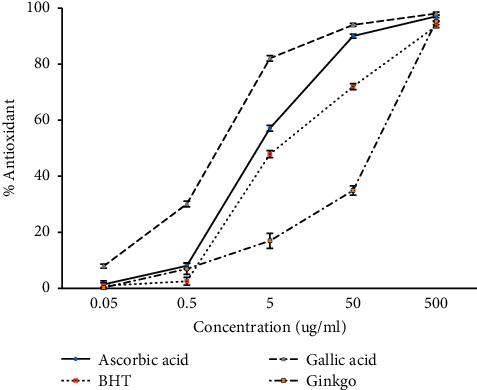
Antioxidant activity of *G. biloba* tea extract with different concentrations compared with that f positive controls ascorbic acid, BHT, and gallic acid (mean ± SD, *n* = 3).

The percent yield of *G. biloba* tea extract was about 17.6% when compared in dry weight condition. Ethanolic extract showed the amount of total phenolic compounds and flavonoid content were 14.13 mg GE g^−1^ DW and 71.33 mg rutin equivalence/g DW, respectively (Table 1). As antioxidant activity is affected by the type and polarity of the solvent, the isolation processes, the integrity of the active compounds, and the system design, the varied antioxidant properties of the phenolic extracts can be ascribed to the varying extracting solvents [23]. Antioxidant activity of all samples (positive control and *G. biloba* tea extract) at lower concentrations has trended to less than that at higher concentrations. At the concentrations of 0.05 and 0.5 *µ*g ml^−1^, BHT, gallic acid, and *G. biloba* tea extract showed the same level of antioxidation, but *G. biloba* tea extract had a significantly lower level than the other two compounds at the concentrations of 5 and 0.5 *µ*g ml^−1^. However, at the highest concentration of the experiment (500 *µ*g ml^−1^), all positive controls and *G. biloba* tea extract did not have a significant difference in the antioxidant activity (Figure 1). The DPPH radical scavenging assay is a useful method for antioxidant research. Scavenging free radicals can inhibit the oxidation reaction of the melanin formation process and is a way of skin whitening [15]. Plant phenolics are a prominent class of secondary plant metabolites having bioactive potential due to antioxidant properties. Depending on seasonal, genetic, and agronomic variables, plant tissue phenolic content varies greatly [24]. Both flavonoid and phenolic compounds are known to have diverse biological activities and may also be responsible for the pharmacological actions or, at least, for synergistically reinforcing those actions [25].

### 3.2. Tyrosinase Inhibition and Anti-DOPA Auto-Oxidation Activity

**Figure 2:**
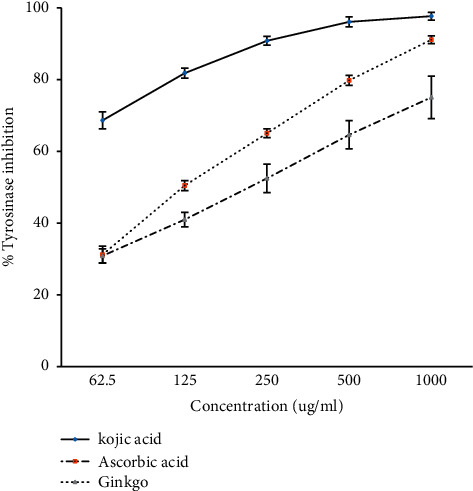
Tyrosinase inhibition of *G. biloba* tea extract with different concentrations compared with that of positive controls kojic acid and ascorbic acid (mean ± SD, *n* = 3).

**Figure 3:**
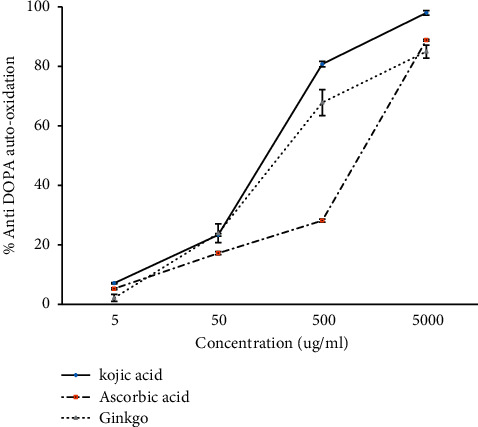
Anti-DOPA auto-oxidation of *G. biloba* tea extract with different concentrations compared with that of positive controls kojic acid and ascorbic acid (mean ± SD, *n* = 3).

Tyrosinase is known as a key enzyme in melanin biosynthesis, which is involved in determination of mammalian skin and hair color. In addition, tyrosinase also promotes enzymatic browning of plant-derived foods, lowering their nutrient content and causing economic damage [26]. The concentration-dependent increase was found in the inhibition of tyrosinase activity. Kojic acid had the highest inhibitory activity at all concentrations. On the other hand, ascorbic acid and *G. biloba* tea extract at the concentration of 62.5 *µ*g/ml did not have a significant difference in the tyrosinase inhibition activity; but, from 125 to 1000 *µ*g ml^−1^ of the concentration, *G. biloba* had the lowest tyrosinase inhibition activity (Figure 2). Kojic acid, ascorbic acid, and *G. biloba* ethanolic extract at the concentrations of 5 and 50 *µ*g ml^−1^ showed the same level of inhibition (no significant difference), whereas, at the concentration of 500 *µ*g ml^−1^, kojic acid had the highest anti-DOPA auto-oxidation followed by *G. biloba* tea extract and ascorbic acid with significant differences, respectively. Furthermore, at the concentration of 5000 *µ*g ml^−1^, the inhibition activity of ascorbic acid and *G. biloba* tea extract was not significantly different, but both of which are significantly less than the activity of kojic acid (Figure 3).

### 3.3. IC_50_

**Figure 4:**
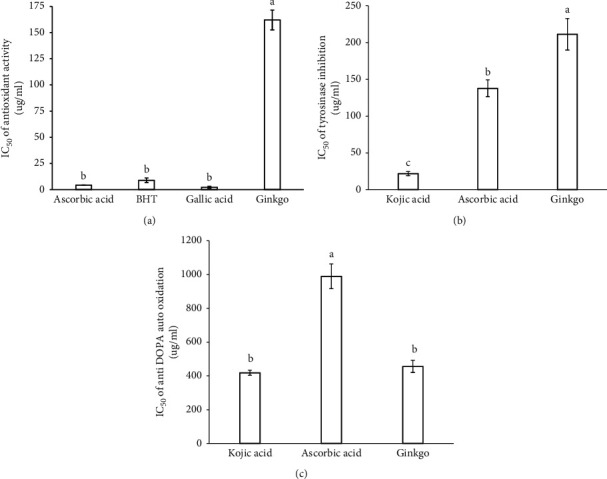
IC_50_ of (a) tyrosinase inhibition, (b) antioxidant activity, and (c) anti-DOPA auto-oxidation. Kojic acid, ascorbic acid, BHT, and gallic acid are used as positive controls. Each bar is presented as mean ± SD (*n* = 3). Different letters indicate significant difference (*p* ≤ 0.05).

Antioxidant activity in terms of IC_50_ showed that the positive controls including ascorbic acid, BHT, and gallic acid have the same level of activity but significantly greater than *G. biloba* tea ethanolic extract (Figure 4(a)). Phenolic compounds may contribute directly to the antioxidative effect of the extracts. Turkoglu et al. [27] reported that indicated that high polyphenolic content was significantly associated with antioxidant activity. *G. biloba* extract had the highest IC50 of tyrosinase inhibition, followed by ascorbic acid, and kojic acid had the lowest, with significant differences (Figure 4(b)). However, kojic acid and *G. biloba* tea ethanolic extract had no significant difference in the IC_50_ of anti-DOPA auto-oxidation and both of which are lower than the IC_50_ of anti-DOPA auto-oxidation of ascorbic acid with statistical significance (Figure 4(c)). The ethanolic extract of *G. biloba* showed biological inhibitory effect including antioxidant, tyrosinase inhibition, and anti-DOPA auto-oxidation that have the inhibition concentration at 50% (IC_50_) of 162.07, 211.91, and 456.27 *µ*g ml^−1^, respectively (Table 2). However, the scavenging of oxygen free radicals is not the only probable mechanism of skin whitening. There are other mechanisms of skin whitening, such as metabolic whitening and anti-inflammatory whitening. Therefore, some plant extracts exhibit good antioxidant activity, while they are with low or without tyrosinase inhibitory activity, for example, *Dioscorea opposita* Thunb [13]. *G. biloba* tea extract is a good source of phenolics. The ethanolic extract of *G. biloba* tea showed biological inhibitory effect including antioxidant, tyrosinase inhibition, and anti-DOPA auto-oxidation; there was no significant difference in the IC_50_ of anti-DOPA auto-oxidation when compared with the positive control kojic acid. *G. biloba* tea should be considered for therapeutic approach in auto-oxidation stress.

### 3.4. Phytochemical Profiling

Phytochemical profiling was done to investigate the presence of medicinally important phytochemicals in *G. biloba* tea. Tannins, flavonoids, terpenoids, and reducing sugars were present in the extract (Table 3), while saponins, steroids, and anthraquinones were absent. Phenolic compounds play an important role in antioxidant activity. Furthermore, the significant antioxidant activity can be due to tannins, flavonoids, terpenoids, and reducing sugars [28].

### 3.5. HPLC Analysis

**Figure 5:**
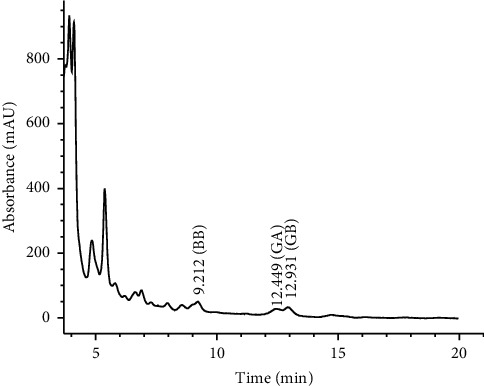
Chromatogram of *G. biloba* tea extract.

HPLC analysis was carried out to analyze the terpenes in *G. biloba* tea extract. The pharmacologically active components, ginkgolide A, ginkgolide B, and sesquiterpene bilobalide, are present in *G. biloba*. A typical chromatogram showed that bilobalide, ginkgolide A, and ginkgolide B were obtained with retention times of 9.2, 12.4, and 12.9 min, respectively (Figure 5). Diterpenes and sesquiterpenes have been proved to be useful in the treatment of allergic diseases, blood disturbances, and neoplastic and immunological disorders [29]. The commercial of *G. biloba* leaf extracts can vary in chemical analysis depending on many factors, e.g., strain, climate, growth phase, and processing. Thus, the analysis of active chemical constituents is important in production of *G. biloba* process control [4, 30].

## 4. Conclusions

This study investigated the effects of antioxidant activity and tyrosinase inhibition of *G. biloba* tea extract. The results revealed that the *G. biloba* tea extract has bioactive chemical ingredients that exhibit antioxidant property, tyrosinase inhibition, and anti-DOPA auto-oxidation. *G. biloba* tea has a potential for protecting living bodies against oxidative cell damage from harmful free radicals, and thus *G. biloba* tea may be a good choice for various applications in a nutraceutical or cosmetic ingredients.

## Figures and Tables

**Table 1:** The amount of constituent compounds of *G. biloba* tea extract (mean ± SD, *n* = 3).

Substances	Content	Unit
Crude extract	175.63 ± 1.87	mg g^−1^ DW

Total phenolic compounds	14.13 ± 0.53	mg GE g^−1^ extract

Flavonoids content	71.33 ± 0.34	mg rutin equivalence g^−1^ DW

**Table 2:** Biological activities of *G. biloba* tea extract in terms of inhibited concentration at 50% (IC_50_) (mean ± SD, *n* = 3).

Biological activities	IC_50_ (*µ*g ml^−1^)
Antioxidant	162.07 ± 9.5
Tyrosinase inhibition	211.91 ± 21.41
Anti-DOPA auto-oxidation	456.27 ± 35.74

**Table 3:** Phytochemical constituents of *G. biloba* tea extract.

Phytochemical constituents	Inference
Tannins	+
Saponins	−
Flavonoids	+
Terpenoids	+
Steroids	−
Anthraquinones	−
Reducing sugars	+

(−) = negative test; (+) = positive test.

## Data Availability

The data used to support the findings of this study are included within the article.
